# Acute fibrinous and organising pneumonia following lung transplantation is associated with severe allograft dysfunction and poor outcome: a case series

**DOI:** 10.15172/pneu.2015.6/648

**Published:** 2015-12-01

**Authors:** Keith C. Meyer, Jennifer Bierach, Jeffrey Kanne, Jose R. Torrealba, Nilto C. De Oliveira

**Affiliations:** 19 University of Wisconsin Lung Transplant and Advanced Lung Disease Program, Section of Allergy, Pulmonary, and Critical Care Medicine, Department of Medicine., University of Wisconsin School of Medicine and Public Health, K4/910 Clinical Science Center, 600 Highland Avenue, Madison, Wisconsin 53792-9988 USA; 29 Utica Park Clinic, Tulsa, Oklahoma USA; 39 Department of Radiology, University of Wisconsin School of Medicine and Public Health, Madison, Wisconsin USA; 49 Department of Pathology, University of Texas Southwestern Medical Center, Dallas, Texas USA; 59 Section of Cardiothoracic Surgery, Department of Surgery, University of Wisconsin School of Medicine and Public Health, Madison, Wisconsin USA

**Keywords:** pneumonia, interstitial, acute fibrinous and organising pneumonia, lung transplantation, thoracic

## Abstract

Acute fibrinous and organising pneumonia (AFOP) is a histopathologic variant of acute lung injury that has been associated with infection and inflammatory disorders and has been reported as a complication of lung transplantation. A retrospective chart review was performed for all patients transplanted at the University of Wisconsin Hospital and Clinics from January 1995 to December 2013 (*n* = 561). We identified 6 recipients whose clinical course was complicated by AFOP. All recipients were found to have AFOP on lung biopsy or at post-mortem examination, and 5 of the 6 patients suffered progressive allograft dysfunction that led to fatal outcome. Only 1 of the 6 patients stabilised with augmented immunosuppression and had subsequent improvement and stabilisation of allograft function. We could not clearly identify any specific cause of AFOP, such as drug toxicity or infection. Lung transplantation can be complicated by lung injury with an AFOP pattern on histopathologic examination of lung biopsy specimens. The presence of an AFOP pattern was associated with irreversible decline in lung function that was refractory to therapeutic interventions in 5 of our 6 cases and was associated with severe allograft dysfunction and death in these 5 individuals. AFOP should be considered as a potential diagnosis when lung transplant recipients develop progressive decline in lung function that is consistent with a clinical diagnosis of chronic lung allograft dysfunction.

## 1. Introduction

Acute fibrinous and organising pneumonia (AFOP) appears to be a variant of acute lung injury (ALI) that has characteristics of diffuse alveolar damage (DAD) as well as features of organising pneumonia (OP) [1]. It has a histopathological pattern that is characterised by intra-alveolar fibrin associated with mononuclear cell infiltrates and loose intraluminal connective tissue with a patchy distribution. AFOP lacks the hyaline membranes that are classically seen in the subacute phase of DAD; it does not have intra-bronchiolar fibrosis (as seen in OP), or prominent infiltration with eosinophils (as seen in eosinophilic pneumonia). AFOP has been linked to various conditions including infections, drug toxicity, and occupational exposures.

Lung transplant recipients are at high risk for both infection and lung allograft rejection. The most significant barrier to long-term survival following lung transplantation is susceptibility to chronic lung allograft dysfunction (CLAD), the predominant cause of which is obliterative bronchiolitis (OB) that leads to bronchiolitis obliterans syndrome (BOS). BOS is characterised by airflow obstruction that usually progresses over time, and is the leading cause of allograft loss and recipient death [2,3]. However, allograft pathologies other than BOS/OB have also been reported as a cause of CLAD. These include restrictive allograft syndrome (RAS), which has been associated with DAD as a precursor lesion and leads to a restrictive pattern of allograft dysfunction with a prognosis that is worse than that for CLAD caused by BOS [4,5]. More recently, Paraskeva et al. [6] identified 87 recipients in a cohort of 194 bilateral lung transplant recipients who developed CLAD; 22 of these patients had a restrictive pattern of allograft dysfunction and were found to have AFOP by histopathologic and radiographic criteria. The recipients who developed AFOP had significantly worse post-transplant survival than a cohort of 39 recipients who developed BOS/OB.

We report on 6 lung transplant recipients who developed severe CLAD that was associated with an AFOP histopathologic pattern on lung biopsy and/or autopsy specimens.

## 2. Methods

We retrospectively reviewed the records (prospectively collected and recorded data in the University of Wisconsin Hospital and Clinics lung transplant database) of all lung and heart-lung recipients who were transplanted at our centre from January 1995 to December 2013. None of the recipients had pre-transplant evidence of sensitization. All recipients had routine clinical evaluations that included chest imaging (posterior-anterior and lateral view chest radiograph [CXR]) and spirometry performed at least every 6 months following lung transplant. Transbronchial and, on occasion (if needed), surgical lung biopsies were performed when suspected allograft dysfunction was detected, and the histopathologic findings from all biopsy specimens were also entered into the database.

All recipients were placed on maintenance immune suppression using tacrolimus, mycophenolate, and corticosteroids, and all received prophylactic antiviral therapy for cytomegalovirus and imidazole antifungal prophylaxis.

All lung transplant recipients received surveillance bronchoscopies with bronchoalveolar lavage (BAL) and transbronchial biopsies (TBBs) to detect acute cellular rejection and/or lymphocytic bronchiolitis with grading according to criteria set by The International Society for Heart & Lung Transplantation (ISHLT) Lung Rejection Study Group [7] at post-transplant weeks 2, 6, 12, 18, 26, 40, and 52. Outpatient clinic evaluations, routine chest imaging, and pulmonary function testing were also performed at these time intervals during the first year.

Clinical evaluations, radiographic imaging, pulmonary function testing, and bronchoscopies were also performed whenever necessary for clinical indications, with follow-up bronchoscopies performed 4 weeks after a previous bronchoscopy where acute rejection of any grade or other significant allograft problem was detected. Thoracic high-resolution computed tomography (HRCT) was obtained if clinically indicated. All biopsy results, thoracic imaging, BAL culture data, and pulmonary function test results were retrospectively reviewed for this study. Screening for donor specific antibodies was not conducted at the time when these recipients were diagnosed with AFOP.

### 2.1 Ethics statement

This investigation was approved by the University of Wisconsin Human Subjects Committee (Approval number: M-2009-1308).

## 3. Results

**Figure 1:**
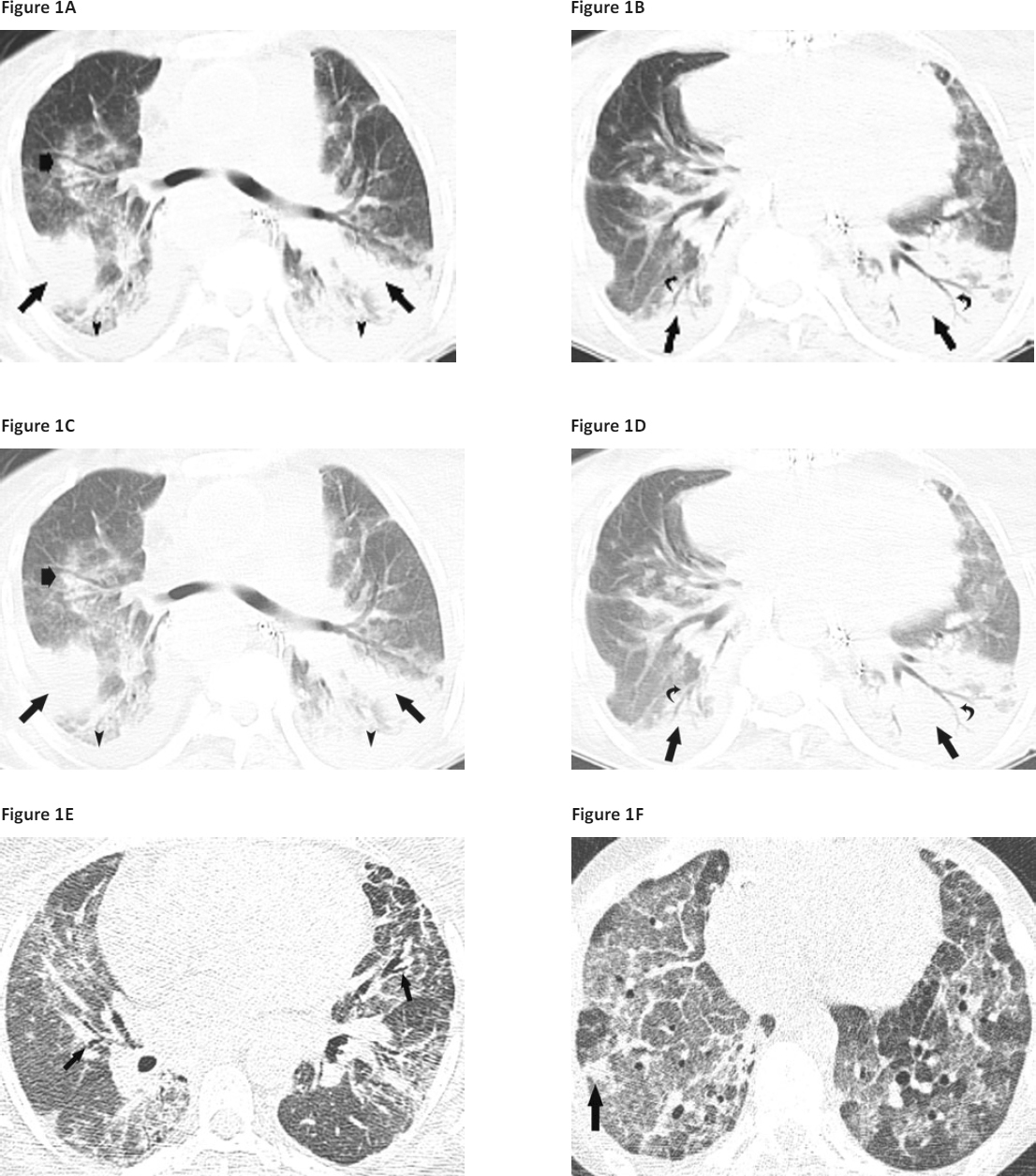
Case 1: Transverse unenhanced computed tomography (CT) images (A and B) demonstrate dense peribronchial consolidation (thin arrows) with air bronchograms (curved arrows); note the rim of consolidation (wide arrow) peripheral to a focus of ground-glass opacity, reminiscent of the “reverse halo” sign seen in organising pneumonia, and small bilateral effusions are present (arrowheads). Case 2: Transverse section (C) shows several foci of peribronchial and perilobular consolidations (arrows) and ground-glass opacity (arrowheads), and small pleural effusions are present. Case 3: Transverse high-resolution computed tomography (HRCT) image (D) demonstrates patchy peribronchial ground-glass opacity with superimposed reticulation and mild traction bronchiectasis (arrows). Case 4: Transverse HRCT images (E and F) show extensive, patchy ground-glass opacity, traction bronchiectasis (arrows in E), superimposed reticulation with peripheral and peribronchial consolation (F), and interlobular septal thickening (F).

The cohort consisted of a total of 561 lung transplant recipients. Of these, 6 recipients were diagnosed as having CLAD due to AFOP. Clinical and demographic characteristics of these 6 patients are given in Table 1, and pertinent data from diagnostic bronchoscopy with BAL and histopathologic findings are given in Table 2. One patient (Case 2) had the diagnosis made via surgical lung biopsy (Figure 1C), and a repeat wedge biopsy from this patient 1 month later showed evidence of persistent AFOP, but prominent polymorphonuclear leukocyte infiltration with fibrin deposition and organising pneumonia (likely due to bacterial infection) was also seen on the second biopsy. The other 5 patients had adequate tissue sampling with TBB that allowed the diagnosis to be made pre-mortem. The diagnosis of AFOP was made at autopsy for Case 3.

**Table 1:** Cohort clinical and demographic data

Case	Age (years)	Gender	Transplant indication and type	Time to onset (days post-transplantation)	Diagnostic modality	Pulmonary function^a^		Treatment	Other complications	Post-transplantation survival (days)
						FEV_1_	FVC			
1	49	Female	CHD with severe PH; HLT	58	TBB	na	na	Antibacterials Corticosteroids	Grade A1B0 acute rejection; *Citrobacter freundii* isolated from blood, BAL and urine	118
2	26	Male	CF; BLT	299	SLB	54%	79%	Antibacterials Corticosteroids	*Candida albicans* and *Stenotrophomonas maltophilia* isolated from blood; MSSA and mucoid *Pseudomonas aeruginosa* isolated from BAL	344
3	41	Female	Fibrotic NSIP; SLT (left)	56	TBB autopsy	97%	88%	Antibacterials Corticosteroids	Persistent poor graft function from the time of transplant; Grade A2B0 acute rejection (previous TBB)	278
4	34	Female	Bleomycin-related PF; BLT	2,572	TBB	70%	72%	Corticosteroids Antibacterials	Multi-drug-resistant *S. maltophilia* isolated from BAL; breast carcinoma metastatic to lungs	2,793
5	71	Female	IPF; SLT (right)	1,354	TBB	63%	74%	Corticosteroids Antibacterials	Onset preceded by symptomatic GER episode	1,514
6	23	Male	CF; BLT	804	TBB	49%	49%	Corticosteroids Antibacterials	URI symptoms and dyspnoea noted for 4 weeks prior to diagnosis	1,476

BAL, bronchoalveolar lavage; BLT, bilateral lung transplant; CF, cystic fibrosis; CHD, congenital heart disease; FEV_1_, forced expiratory volume in one second; FVC, forced vital capacity; HLT, heart-lung transplant; IPF, idiopathic pulmonary fibrosis; MSSA, methicillin-sensitive *Staphylococcus aureus*; na, not able to perform; NSIP, non-specific interstitial pneumonia; PF, pulmonary fibrosis; PH, pulmonary hypertension; SLB, surgical lung biopsy; SLT, single lung transplant; TBB, transbronchial lung biopsy; URI, upper respiratory infection; GER, gastro-oesophageal reflux

^a^percentage of stable baseline values (average of 2 best results prior to acute fibrinous and organising pneumonia [AFOP] onset) at time of AFOP diagnosis

**Table 2:** Bronchoalveolar lavage (BAL) fluid analysis and transbronchial lung biopsy (TBB) findings (performed when acute fibrinous and organising pneumonia [AFOP] was diagnosed)

Case	BAL									TBB grade
	Cells/µl	Neutrophils (%)	Lymphocytes (%)	Eosinophils (%)	Macrophages (%)	Microbiology				
						Gram stain	Bacterial culture		Other^a^	
							Bacteria	Concentration (cfu/µl)		
1	2,130	69	4	1	23	No bacteria	Oral flora	<10^3^	None	A0B0 AFOP
2	875	48	0	0	6	Bacteria present	MSSA mucoid *Pseudomonas aeruginosa*	4 × 10^4^ 3 × 10^4^	None	A0B0 AFOP
3	295	15	53	0	30	No bacteria	NG		None	A0B0 AFOP
4	330	44	27	1	26	No bacteria	*Stenotrophomonas maltophilia*	8 × 10^3^	*Aspergillus fumigatus* (Galactomannan negative)	A0B0 AFOP
5	188	28	16	4	49	No bacteria	NG		Cytomegalovirus PCR positive	A0B0 AFOP
6	200	10	38	8	40	Rare bacteria	MSSA	2 × 10^4^	None	A1B1 AFOP

cfu, colony forming units; MSSA, methicillin-sensitive *Staphylococcus aureus*; NG, no growth; PCR, polymerase chain reaction

^a^All BAL specimens were evaluated (stains/cultures/PCR) for *Pneumocystis jiroveci* and other fungi, bacterial pathogens (quantitative), viruses (cytomegalovirus, herpes simplex, respiratory syncytial virus, influenza, parainfluenza, metapneumovirus, rhinovirus, and adenovirus), mycobacteria, and *Nocardia* spp.

## 4. Case series

### 4.1 Case 1

**Figure 2:**
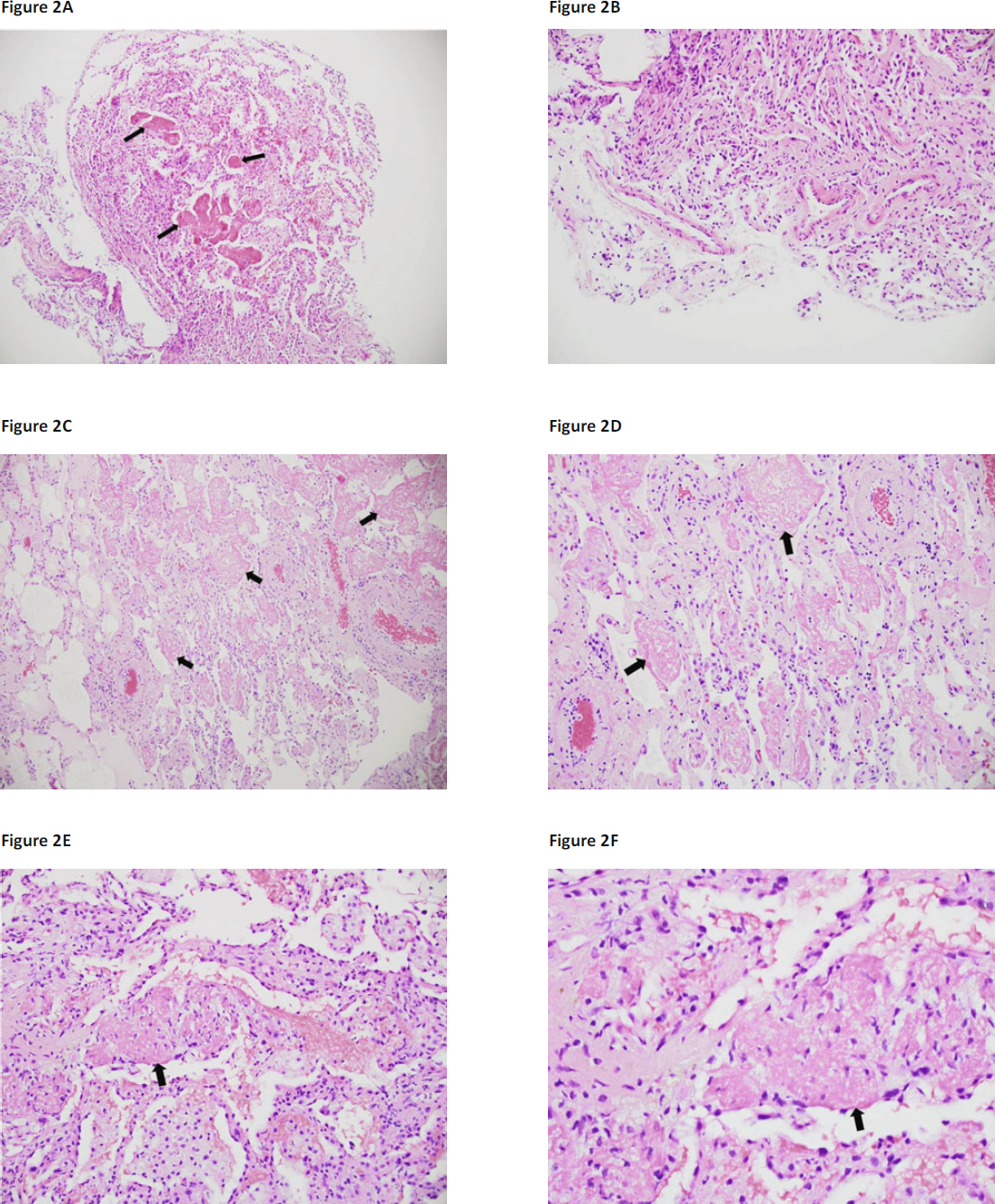
Case 1: Transbronchial biopsy shows lung parenchyma with intra-alveolar fibrin aggregates or “balls” (arrows) with mild mononuclear interstitial infiltrate (A: Haematoxylin-eosin stain [H/E], 100× magnification), without evidence of cellular rejection given the lack of perivascular or bronchial mucosal infiltrates (B: H/E, 200× magnification). Case 2: Surgical lung biopsy shows intraalveolar fibrin aggregates (arrows) and mild fibroblastic proliferation with minimal mononuclear inflammatory cell infiltrate (C: H/E 100× magnification; D: H/E 200× magnification). Case 3: Autopsy specimen shows intra-alveolar fibrin aggregates (arrows) (E: H/E 100× magnification; F: H/E 200× magnification).

A 48-year-old female received a heart-lung transplant for congenital heart disease. Early sputum cultures demonstrated Gram-negative rods for which piperacillin-tazobactam was given, and she was extubated on the second day, post-transplant, and commenced ambulation. However, small bowel complications developed and required surgical resection, and she developed dialysis-dependent acute renal insufficiency. Intubation and mechanical ventilation were required on day 30, and chest imaging showed persistent diffuse infiltrates (Figure 1, A and B). TBB showed minimal acute rejection (Grade A1B0). She was extubated 9 days later but required reintubation (post-transplant day 58) for progressive respiratory failure. TBB biopsy showed AFOP but did not show acute rejection (Figure 2, A and B), and an endomyocardial biopsy was negative for cardiac rejection. A laparotomy and right hemicolectomy were performed for persistent volvulus (post-transplant day 100). Lung function worsened, and *Citrobacter freundii* was isolated from blood cultures, BAL, and urine cultures. Respiratory status progressively worsened, and life support was withdrawn.

### 4.2 Case 2

A 26-year-old man received a successful bilateral lung transplant for end-stage cystic fibrosis but required stent placement for a right main bronchial stenosis at the anastomosis site. He developed cough, dyspnoea, and fever 5 months post-transplantation; sputum cultures demonstrated mucoid *Pseudomonas aeruginosa* for which piperacillin-tazobactam was administered. TBB showed minimal acute rejection (Grade A1B0). Cough and dyspnoea persisted and the patient resumed use of supplemental home oxygen. He acutely decompensated 2 weeks later with diffuse infiltrates on thoracic imaging (Figure 1, C) and developed shock and respiratory insufficiency, requiring intensive care unit admission and administration of pressors, tracheal intubation, and mechanical ventilation. Group A *Streptococcus pyogenes* was present in multiple blood cultures, and diabetic ketoacidosis was also present. He was successfully treated, extubated, and discharged to home 12 days after admission. After a 3-month period of clinical stability, he developed sustained decline in forced expiratory volume in 1 second (FEV_1_). BAL showed methicillin-sensitive *Staphylococcus aureus* (MSSA) and mucoid *P. aeruginosa*. TBB did not show acute rejection or other specific parenchymal abnormality. Antibiotics were administered, and blood cultures grew *Candida albicans* and *Stenotrophomonas maltophilia*. Intubation and mechanical ventilatory support were required. Due to lack of improvement with appropriate antibacterial and anti-fungal therapies, a surgical lung biopsy was performed that showed AFOP (Figure 2, C and D). Chest imaging showed persistent infiltrates, and care was withdrawn after 1 month of mechanical ventilation.

### 4.3 Case 3

A 41-year-old female received a left single lung transplant for fibrotic non-specific interstitial pneumonia (NSIP). At 2 weeks post-transplant surveillance, TBB specimens showed Grade A1B1 acute rejection, and TBB performed 2 weeks later showed Grade A2B0 acute rejection. Intravenous methylprednisolone burst and taper was given, and she remained asymptomatic and continued to improve clinically until 6 weeks post-transplant when she presented with increasing dyspnoea on exertion, cough, and low-grade fever that progressed to intubation and mechanical ventilation. She was treated with broad-spectrum antibacterial agents and pulse corticosteroids. TBB demonstrated organising pneumonia (OP) but no acute rejection, and BAL did not reveal any pathogens. She was successfully extubated after 4 days, weaned to room air, and discharged to home. However, subsequent surveillance TBBs showed persistent OP as well as acute rejection (Grade A2–A3), and total lymphoid irradiation was initiated. The patient was readmitted 6 weeks later for shortness of breath and hypoxaemia that progressed to respiratory failure, requiring mechanical ventilation. Despite broad-spectrum antibiotics and pulse corticosteroids, diffuse parenchymal opacities (Figure 1, D) progressively worsened, and supportive care was eventually withdrawn. AFOP was observed in lung specimens examined at autopsy (Figure 2, E and F).

### 4.4 Case 4

A 34-year-old woman who had been considered cured of Hodgkin’s lymphoma underwent bilateral lung transplantation for bleomycin-associated pulmonary fibrosis. She had a benign clinical course for the next 7 years despite persistent poor graft function, but was then diagnosed with a left breast ductal carcinoma and underwent mastectomy and was started on tamoxifen. Three months later she noted persistent mild symptoms of cough and dyspnoea on exertion, associated with new persistent infiltrates on imaging (Figure 1, E and F), accompanied by a significant decline in FEV_1_ and oxyhaemoglobin desaturation with activity. TBB revealed AFOP, and high-dose corticosteroids and broad-spectrum antibiotics were given. BAL detected multi-drug resistant *S. maltophilia*, for which antibacterial coverage was adjusted. She gradually improved and was discharged to home, but after a stable period of 6 months, she developed increased shortness of breath, and a thoracic HRCT showed multiple new lung nodules. Lung biopsy revealed metastatic breast cancer, to which she succumbed 3 months later.

### 4.5 Case 5

A 67-year-old female received a right single lung transplant for idiopathic pulmonary fibrosis and had an uncomplicated post-transplant course; surveillance bronchoscopies did not show acute rejection or infection, and 4 months post-transplantation her FEV_1_ reached a zenith of 129% of predicted. She remained stable for the next 44 months until diagnosed with BOS (Stage 1). Chest imaging revealed new patchy consolidation in the allograft. Bronchoscopy revealed BAL neutrophilia (28%), and cytomegalovirus was detected in BAL (but not peripheral blood) via polymerase chain reaction. TBB was negative for acute rejection or cytomegalovirus pneumonitis, but showed changes of AFOP. Intravenous pulse methylprednisolone and antibiotics were administered with gradual clearing of the allograft infiltrates on CXR. However, FEV_1_ continued to decline, and follow-up bronchoscopy 2 months later revealed persistent AFOP and BAL neutrophilia (56%), despite clearing of the allograft infiltrates on CXR. BAL was negative for infection. FEV_1_ subsequently progressively declined and the patient eventually succumbed to respiratory failure.

### 4.6 Case 6

A 22-year-old male received a bilateral lung transplant for cystic fibrosis and had an essentially uncomplicated post-transplantation course for 26 months (with the exception of a Grade A2 rejection episode at 12.5 months post-transplant). He developed worsening cough, increasing fatigue, and dyspnoea following exposure to his spouse who had developed upper respiratory tract infection symptoms. Spirometry showed a significant decline in FEV_1_, and oxyhaemoglobin desaturation was noted. TBB revealed changes of AFOP and was also graded as showing a Grade A1B1 acute rejection, and BAL cultured MSSA at 2 × 10^4^ colony forming units (cfu)/µl but was negative for respiratory viruses. The patient’s symptoms initially improved with antibiotics and pulse methylprednisolone, but FEV_1_ subsequently progressively declined and the patient eventually succumbed to respiratory insufficiency.

## 5. Discussion

Beasley and colleagues [1] described AFOP as a new histopathologic entity in 2002 and concluded that it represented an unusual variant of ALI. OP is characterised by buds of granulation tissue (comprised of fibroblasts and myofibroblasts in a matrix of loose connective tissue) in distal airspaces (alveolar ducts, alveoli, and bronchioles) that may be idiopathic (cryptogenic organising pneumonia [COP]) [8,9]. OP has been associated with a variety of clinical entities such as infection, connective tissue disorders, or pneumotoxic drug reactions [9]. DAD has also been associated with a number of clinical disorders such as infection or drug toxicity; it is characterised by alveolar epithelial cell and endothelial injury with fluid and cellular exudation followed by reparative fibroblast proliferation and alveolar type II pneumocyte hyperplasia [10]. Hyaline membrane formation marks the exudative phase of DAD. AFOP is characterised histopathologically by intra-alveolar fibrin (in the form of fibrin “balls”) within the alveolar spaces [11]. The presence of the fibrin balls differentiates AFOP from classical OP (with its granulation tissue in the bronchioles, alveolar ducts, and alveoli) as well as from DAD (with its classic hyaline membranes). Patchy distribution of fibrin deposition is typical and involves 25–90% of alveolar spaces, averaging 50% of airspace involvement overall. In contrast to AFOP, DAD is typically diffuse. Although AFOP lacks the classic hyaline membranes that characterise DAD, myxoid fibroblastic tissue has been identified in the alveolar walls in both AFOP and DAD. Eosinophils are typically present, but not predominant as with eosinophilic pneumonitis.

Routine chest radiographs of patients with AFOP typically show interstitial changes and may show areas that appear consolidated [1,12]. All of our patients had diffuse, persistent infiltrates on thoracic imaging. HRCT imaging (Figure 2) showed peribronchial and perilobular consolidation with areas of ground-glass opacity. Bronchoalveolar lavage performed at the time of diagnosis of AFOP (Table 2) showed considerable BAL neutrophilia for all patients, but only 2 patients had bacteria visualised on Gram stain at ≥1 × 10^4^ cfu/µl of BAL fluid.

Many clinical conditions have been associated with AFOP, and AFOP has been linked to drug reactions (e.g. decitabine, amiodarone, abacavir), infections (e.g. severe acute respiratory syndrome, respiratory syncytial virus, *Haemophilus influenzae, Acinetobacter*), and various forms of connective tissue disorders [1,13,14]. Other associations include stem cell transplantation, haematologic malignancy, occupational exposures, and cigarette smoking [13,14]. Although AFOP often runs a rapid and fatal course, responses to pharmacologic interventions have been reported. Treatments associated with significant clinical responses include corticosteroids, cyclophosphamide, and mycophenolate [15]. Acute onset accompanied by the need for mechanical ventilation appears to correlate with a poor prognosis [1].

Infection and/or drug toxicity may have played a key role in AFOP pathogenesis in our patients. Another possibility is that AFOP represented a form of allograft rejection, although these patients had ongoing intense immunosuppression and various complications associated with their lung transplant status, and 3 of the 6 patients did not have improvement with intensified immune suppression. None of our subjects received drugs that have been linked to AFOP in published literature. Additionally, none of the surgical or TBB specimens revealed a consistent association with acute rejection or lymphocytic bronchiolitis. Because of the intense immunosuppression and multiple drugs that our lung transplant recipients were receiving, plus the numerous drugs (many associated with potential pulmonary toxicity) they were taking for management of their post-transplant issues as well as ongoing colonisation and/or infection with potential pathogens, it is difficult to associate AFOP with any specific cause. Only 1 of our 6 patients stabilised and appeared to recover. Her dyspnoea, infiltration on chest radiographs, and gas exchange were rapidly worsening, but she stabilised and gradually improved when intensified immunosuppression (intravenous methylprednisolone and subsequent gradual prednisone taper) was given. A point of interest is the infrequent occurrence of AFOP in our recipient population (6 of 561 [1%]) as compared to the report by Paraskeva et al. (22 of 194 [11%]) [6].

Sato et al. [4] used a ≥10% decline in baseline total lung capacity (TLC) to indicate the presence of restrictive physiology in addition to the presence of radiographic infiltrates on chest imaging and DAD on histopathology as diagnostic of the RAS form of CLAD. Although we did not measure lung volumes in our cohort of recipients with AFOP, the clear decline of both FEV_1_ along with forced vital capacity (FVC) in 4 of the 6 subjects is consistent with restrictive physiology. Because most centres do not measure lung volumes and many factors can confound measurements of TLC, we suggest that the combination of spirometry, imaging findings, and histopathology can be used to identify AFOP and differentiate it from the RAS or the classical obstructive BOS/OB phenotypes of CLAD [3]. Our experience plus that reported by Paraskeva et al. [6] indicates that AFOP should be considered as a diagnosis in transplant recipients who develop allograft dysfunction when clinical evaluation suggests that CLAD associated with restrictive physiology and radiographic infiltrates has developed. Although upper lobe infiltrates in this setting are highly suggestive of a diagnosis of RAS [4,6], lung biopsy is required to differentiate AFOP from RAS.

In conclusion, we identified 6 lung transplant recipients who developed diffuse infiltrates and had histopathologic changes of AFOP in lung tissue specimens, indicating that AFOP can be associated with lung function decline following transplantation. Although AFOP has been associated with a number of inciting factors in non-transplant patients, we could not definitively link a specific cause of declining lung allograft function with the diagnosis of AFOP for any of our patients. However, 3 of our recipients had serious infectious complications and/or episodes of allograft rejection prior to or during the onset of AFOP. We conclude that histopathologic changes consistent with AFOP can bet associated with persistent post-transplant decline in allograft function; that recipients who develop AFOP may meet criteria that are consistent with a diagnosis of CLAD with clinical characteristics that can overlap with the RAS subset of CLAD, (infiltrates on HRCT, decline in FVC consistent with ventilatory restriction); that it occurs infrequently; and that its appearance is associated with a poor prognosis. Further characterisation of the AFOP phenotype of CLAD may assist efforts to prevent and/or treat this potential complication of lung transplantation.
